# Metabolic health assessment of zoo elephants: Management factors predicting leptin levels and the glucose-to-insulin ratio and their associations with health parameters

**DOI:** 10.1371/journal.pone.0188701

**Published:** 2017-11-29

**Authors:** Kari A. Morfeld, Janine L. Brown

**Affiliations:** 1 Lincoln Children’s Zoo, Lincoln, Nebraska, United States of America; 2 Center for Species Survival, Smithsonian Conservation Biology Institute, Smithsonian National Zoological Park, Front Royal, Virginia, United States of America; University of Tasmania, AUSTRALIA

## Abstract

Screening for metabolic-related health problems can enhance animal welfare, so the purpose of this study was to conduct the first metabolic health assessment of zoo elephants and use epidemiological methods to determine how factors in the captive environment were associated with metabolic hormone concentrations. In addition, we examined relationships between metabolic status and several fitness parameters: foot health, musculoskeletal health, reproductive cyclicity, and body condition. Two blood samples were collected 2 weeks apart from 87 Asian (*Elephas maximus*) and 105 African (*Loxodonta africana*) elephants managed by zoos accredited by the Association of Zoos and Aquariums for analysis of serum leptin, insulin, glucose and the glucose-to-insulin ratio (G:I). In females, mean (± SD) leptin concentrations and the G:I were lower (P<0.05) in Asian (3.93 ± 2.21 ng/ml and 110 ± 86 units) compared to African (4.37 ± 2.89 ng/ml and 208 ± 133 units) elephants, respectively. For males, mean leptin and the G:I were 4.99 ± 3.61 ng/ml and 253 ± 181 units for Asian, and 3.72 ± 2.00 ng/ml and 326 ± 231 units for African elephants, respectively, with no differences between species (P>0.05). As mean leptin concentration increased there was an increase in the odds of a female being non-cycling (P = 0.0083). The G:I was associated inversely with body condition (P = 0.0002); as the G:I increased there was a decreased risk of BCS = 4 or 5 as compared to the ideal, or BCS = 3. Neither leptin nor G:I were predictive of foot or musculoskeletal health scores. Factors related to walking and feeding practices were most influential in predicting metabolic status, whereas social and housing factors showed smaller, but significant effects. The metabolic health benefits of walking were detected if the time spent in staff-directed walking was 7 hours or more per week. The most protective feeding practices included implementing a random rather than predictable feeding schedule and limiting the number of methods presentation methods. Results indicate that leptin levels and G:I can be used as predictors of both ovarian cycle function and body condition, and are affected by zoo management in elephants.

## Introduction

Metabolic health is important for physical well-being, and can be assessed by measuring physiological indicators involved in energy balance such as glucose, insulin and leptin. Prolonged elevation of blood glucose has toxic effects on multiple cell types [1], and therefore needs to be maintained within narrow limits by precise hormone regulation, primarily by insulin. Insulin is secreted in response to rising blood glucose levels, and acts to increase glucose transport, metabolism, and storage in muscle and adipose tissue [2]. Insulin resistance results when there is a diminished ability of cells to respond to the action of insulin in transporting glucose from the bloodstream into muscle and other tissues. Measures of glucose and insulin are generally made after a patient has fasted, but a glucose-to-insulin ratio (G:I) can be used instead and often serves as a proxy for counteracting the effects of glucose and/or insulin changes due to feeding status [3]. Leptin, mainly produced in adipose tissue, is an indicator of body energy status. It contributes to satiety and the regulation of food-intake, acting on the hypothalamus to reduce appetite [4], with circulating levels proportional to fat mass in several species [5–9].

Aberrant metabolic hormone function is an underlying driver of many health problems and diseases in a number of species. For example, obesity, metabolic syndrome, diabetes, and infertility are associated with dysregulation of insulin and glucose in humans [10–15]. In horses, insulin resistance is related to metabolic syndrome [16, 17], Cushing’s disease [18], laminitis [19, 20] and infertility [21]. In humans, hyperleptinemia is observed in obesity, type 2 diabetes, metabolic syndrome, chronic renal failure and atherosclerosis [22, 23], and can be predictive of future cardiovascular events [24]. Leptin exerts its effects on the immune system and participates in the pathogenesis of many autoimmune inflammatory processes, such as diabetes, rheumatoid arthritis and psoriasis [25, 26]. Consequently, the monitoring of metabolic hormones has become paramount in screening for and management of metabolic-related disorders.

Despite the recognition that metabolic hormones are involved in many disease states, surprisingly little research has been conducted to assess their role in elephant health. Commonly reported health concerns causing morbidity and mortality in zoo elephants include foot and musculoskeletal conditions [27–30], reproductive acyclicity [31–33], and obesity [34–37] each of which could be related to metabolic status. Recently, Morfeld and Brown [36] found significant relationships between reproductive function and metabolic activity in African elephants, with non-cycling elephants having higher concentrations of insulin and leptin and a lower G:I ratio. That study was the first to document a link between metabolic and reproductive health in elephants, setting the stage for further investigations on the role of metabolic hormones and associations with other health issues affecting zoo elephants. The objectives of this study were to: 1) assess the metabolic health status of male and female Asian and African elephants in AZA zoos through analyses of glucose, insulin and leptin; 2) test for associations between metabolic hormones and several health parameters: foot health, musculoskeletal health, reproductive cyclicity, and body condition; and 3) investigate the associations of demographic, management, housing, and social variables to determine their impact on elephant metabolic function. Our goal is to understand how the zoo environment affects metabolic health status of zoo elephants, information that can be used to drive management decisions that optimize elephant health, reproduction and welfare.

## Materials and methods

### Ethics statement

All data included in this study were sourced from elephant programs at zoos accredited by the AZA and enrolled in the Using Science to Understand Zoo Elephant Welfare study [38]. These zoos were located in the United States, Mexico and Canada. This study was authorized by the management at each participating zoo and, where applicable, was reviewed and approved by zoo research committees. In addition, the study protocol was reviewed and approved by the Zoological Society of San Diego Institutional Animal Care and Use Committee N.I.H. Assurance A3675-01; Protocol 11–203. Approval also was obtained from the Smithsonian National Zoo ACUC (#11–10).

### Animals and blood sample collection

The study population was comprised of 87 Asian (n = 18 male and n = 69 female) and 105 African (n = 23 and n = 82 female) elephants at 69 American Zoo and Aquarium accredited zoos. The mean age of the Asian elephants was 37.2 years (range = 5 to 61) and the mean age of African elephants was 28.4 years (range = 1 to 52). Two blood samples collected two weeks apart between October and December 2012 were used to assess metabolic hormone levels of each study elephant. The coefficients of variation were <15% between the two samples to account for potential sample variation within individuals. Reproductive cyclicity was determined by analysis of serum progestogens over a 12-month period from bi-weekly blood samples as reported by Brown et al. [32]. Blood was collected without anesthesia from either an ear or leg vein; protocols requested blood draws to occur before 12 noon. Blood was allowed to clot at room temperature and centrifuged at ~1500g for 15 min within 3 hours of collection. Serum samples were stored at -20°C or colder until analysis.

### Health and reproductive parameters

The health parameters investigated for associations with metabolic hormones were a subset of those evaluated in conjunction with a national welfare study [38] as described previously: foot and musculoskeletal health [30], reproductive cyclicity [32], and body condition [37]. A physical examination was conducted by zoo-based veterinarians on each elephant about mid-way during the study period in conjunction with collection of the two blood samples for metabolic analyses. Foot health was assessed using a standard template, and each toenail was evaluated for cracks, defects, or horn growth abnormalities. Incidences of cracks, ulcerations, abscesses, or growth on the foot pads or in the interdigital spaces were also recorded. Each elephant was assigned a score based on the following system: each of three locations (toenail, pad, or interdigital space) on a foot were examined for an abnormality, and each location on each foot with an abnormality was scored as 1, such that each foot could have a maximum score of 3, with each elephant having a maximum score of 12. The musculoskeletal (MS) condition of each elephant was evaluated using a standard template to assess the condition of each joint (shoulders, elbows, carpi, hips, stifles, tarsi). Abnormalities documented included: swelling, heat, calluses, abscess, fistula, or deformity, and range of motion of each joint was evaluated for stiffness or mechanical limitations. Each elephant was assigned a MS health score based on the following system: a MS health score of 0 indicated no abnormal range of motion (ROM) or joint abnormalities noted on the physical exam. A score of 1 indicated one abnormal joint or ROM, a score of 2 indicated one abnormal joint and one abnormal ROM, and a score of 3 indicated two or more abnormal joints and abnormal ROM.

To assess body condition, participating zoos were provided a photographic guide containing detailed instructions on how to obtain three standardized photos for each elephant for visual body condition assessment. The visual BCS methods were validated for both African [35] and Asian [37] elephants and consisted of a list of key body regions (ribs, backbone, and pelvic bone) and the physical criteria used for assigning an overall score on a 5-point scale, with 1 representing the lowest and 5 representing the highest levels of body fat.

Females were categorized as normal ovarian cycling (regular 13- to 17-week cycles), irregular cycling (follicular or luteal phases that were longer or shorter than normal), or acyclic (baseline progestogens, <0.1ng/ml throughout) [32].

### Hormone analyses

Serum leptin and insulin were analyzed using immunoassays previously validated for use in elephants [36]. Briefly, serum leptin concentrations were measured using a multi-species double-antibody radioimmunoassay (RIA) (Xl-85K, Linco Research Inc., St. Louis, MO, USA) that relies on a ^125^I-human leptin tracer and a guinea pig anti-human leptin antiserum. Serum insulin concentrations were measured using a solid-phase, two-site bovine insulin enzyme immunoassay (EIA) (10-1113-01, Mercodia Inc., Uppsala, Sweden). Serum glucose was determined using an automated glucose analyzer (One Touch Ultra, LifeScan, Inc., Milpitas, CA, USA) and a G:I was calculated to account for the non-fasting state of our study animals. Serum progestogens were quantified using a solid-phase ^125^I radioimmunoassay (Siemens Medical Solutions Diagnostics, Los Angeles, CA). All samples were analyzed in duplicate; intra- and inter-assay CVs were <10% and <15%, respectively.

### Zoo variables

A variety of independent zoo variables were investigated to assess potential associations with mean leptin levels and G:I. Definitions of each variable are shown in Table 1. Details on the collection and calculation of housing and social variables are presented in Meehan et al. [39], life history related variables are described in Prado-Oviedo et al. [40], and Greco et al. [41] provides background on variables related to exercise, training, feeding practices and environmental enrichment. Feeding Predictability originally consisted of three categories: “predictable” (feeding times were consistent from day to day); “semi-predictable” (feeding times were intentionally varied by up to 60 min from day to day); and “unpredictable/random” (feeding times were not scheduled or occurred randomly), but for this study was converted to a binary variable, with “predictable” and “semi-predictable” combined and classified as “predictable schedule” as the reference category.

**Table 1:** Descriptions of variables tested for associations with leptin concentrations and the glucose-to-insulin ratio.

Variable ^a^	Unit of Analysis	Description
**Demographics** ^**1**^		
Age	Elephant	Age of elephant (years)
Sex	Elephant	Female or male
Species	Elephant	African or Asian
**Exercise** ^**2**^		
Exercise Week	Elephant	Categorical number of reported hours spent exercising animals each week on a seven-point scale ranging from 1 to 7: 1 = < 1 hour, 2 = 1–3 hours, 3 = 4–6 hours, 4 = 5–7 hours, 5 = 7–10 hours; 6 = 10–14 hours; 7 = 14 or more.
Walk Week	Elephant	Categorical number of reported hours spent walking elephants each week on a seven-point scale ranging from 1 to 7: 1 = < 1 hour, 2 = 1–3 hours, 3 = 4–6 hours, 4 = 5–7 hours, 5 = 7–10 hours; 6 = 10–14 hours; 7 = 14 or more.
**Feeding** ^**2**^		
Feed Day	Zoo	Number of feedings during the day
Feed Night	Zoo	Number of feedings during the night
Feed Total	Zoo	Sum of feedings during the day and night
Feeding Predictability	Zoo	The predictability of feeding activities; 1 = predictable: feeding times consistent, and may intentionally vary by up to 60 min, from day to day, and 2 = unpredictable: feeding times are not scheduled and occur randomly
Feed Diversity	Zoo	Shannon diversity index of the number of feeding types and frequency with which each type was provided
Spread	Zoo	Relative frequency of the percentage of time food was spread around the exhibit compared to all feeding techniques
Alternative Feeding Methods	Zoo	Relative frequency of the percentage of time food was presented in a foraging device, hidden, or hanging compared to all feeding types
**Housing** ^**3**^		
Percent Time Indoor	Elephant	Percent time spent in indoor environments
Percent Time In/Out Choice	Elephant	Percent time spent in environments with an indoor/outdoor choice
Space Experience (500 ft^2^)	Elephant	Average size of the environments an elephant spends time in, weighted by the amount of time spent in each environment
Space Experience per Elephant (500 ft^2^)	Elephant	Average size of environments an elephant spends time in, divided by the total number of elephants in social group using the space at that time, weighted by the amount of time spent in each environment
**Social** ^**3**^		
Animal Contact	Elephant	Maximum number of unique elephants focal animal is in contact with
Social Group Contact	Elephant	Maximum number of unique social groups focal animal is part of
**Training and Enrichment** ^**2**^		
Relative Positive Reinforcement and Negative Punishment	Elephant	The relative frequency with which an elephant experienced Positive Reinforcement and/or Negative Punishment over all types of reinforcement and punishment; ranging from 1 (never) to 9 (very frequently)
Enrichment Diversity	Zoo	Shannon diversity index of the number of enrichment types and frequency with which they were provided

^a^ References for variable development and description

^1^Prado-Oviedo et al. [40]

^2^Greco et al. [41]

^3^ Meehan et al. [42].

### Statistical analysis

Data are expressed as mean ± standard deviation (SD). Differences in mean leptin, glucose, and insulin concentrations, and the G:I between species were determined using unpaired t-tests. Generalized linear regression analyses were used to assess mean leptin and G:I as predictors of health outcomes measured in associated studies. Age, sex, and species were tested as potential confounding variables. Descriptive statistics for insulin and glucose are presented, but these hormones were not individually analyzed as predictors of health outcomes due to the non-fasting status of the elephants before blood sample collection. The health outcomes included foot health [30], musculoskeletal health [30], reproductive cyclicity [32] and body condition [37]. Generalized linear regression models to assess these outcomes were specific to the distribution of the outcome. Foot health consisted of a count score of 0–12, and was tested using Poisson distribution, log link, and deviance scale d. Musculoskeletal health consisted of a 0–3 scale associated with presence of abnormal joints or range of motion, and was tested using multinomial logistic and a cumulative logit link function, with 0 as the reference value. Reproductive cyclicity consisted of “Cycling” or “Non-cycling” and was tested using binomial distribution and a logit link function, with “Cycling” as the reference. The “ideal” body condition score of 3 was compared to elevated body condition scores of 4 or 5 using a multinomial logistic distribution and a cumulative logit link function. All regression methods utilized an independent correlation matrix.

Univariate analyses were conducted on all independent variables of interest across all subjects. Variables with a significance level of P<0.10 were tested for possible confounding effects of age, sex, and species and allowed to continue in the hierarchical model building process. Because age, sex, and species could influence both outcome and the tested input variable, these variables were tested as potential confounding variables. Confounding variables (those that altered the beta values of input variables by more than 10% during bivariate analysis) were included in all models. Multi-variable models were then fit using generalized estimating equations, which allows for repeated measurement and clustering of individual animals within zoos. At the multivariate stage, only variables with a significant level of P < 0.05 were included. Models were built by assessing individual predictors and conducting hierarchical selection based on quasi-likelihood under the independence model criterion (QIC) values and parameter estimates of explanatory variables. Models exhibiting multi-collinearity, as defined by a variance inflation factor (VIF) of greater than 10 and a Condition Index (CI) of greater than 30, were not considered for further analysis. The model used an independent correlation matrix type. Statistical analyses were conducted using SAS software, version 9.3 [PROC GENMOD, with options REPEATED, CORR = IND or AR, and DIST = NORMAL; SAS Institute, Inc., Cary, NC].

## Results

### Metabolic status

Mean and range of leptin concentrations according to species and sex are presented in Table 2. There was no difference between the mean leptin concentrations overall between Asian and African elephants (P>0.05), or between species of similar sex (females P = 0.142; males P = 0.096). The lowest leptin value was observed in an African female elephant (0.96 ng/ml), whereas the highest value was noted in an Asian male elephant (13.99 ng/ml). There was a large range in leptin data for males and females of both species.

**Table 2:** Mean ± SD and range of leptin concentrations (ng/ml) in male and female Asian and African elephants.

	Asian			African		
Sex	N	Mean ± SD	Min-max	N	Mean ± SD	Min-max
Female	69	3.93 ± 2.21	1.23–11.88	82	4.37 ± 2.89	0.96–11.99
Male	18	4.99 ± 3.61	1.26–13.99	23	3.72 ± 2.00	1.06–9.24
Overall	87	4.07 ± 2.57	1.23–13.99	105	4.16 ± 2.50	0.96–11.99

Mean and range of glucose and insulin concentrations according to species and sex are presented in Tables 3 and 4, respectively. There was no difference between mean glucose concentrations overall between Asian and African elephants (P>0.05), or between species of similar sex. Mean insulin concentrations were higher in female Asian compared to female African elephants (P<0.001). However, there was no difference in mean insulin concentrations between Asian and African males (P = 0.766).

**Table 3:** Mean ± SD and range of glucose concentrations (mg/dl) in male and female Asian and African elephants.

	Asian			African		
Sex	N	Mean ± SD	Min-Max	N	Mean ± SD	Min-Max
Female	79	101 ± 28	38–181	100	95 ± 21	21–152
Male	13	97 ± 24	56–142	17	98 ± 19	69–135
Overall	92	100 ± 28	38–181	117	96 ± 21	21–152

**Table 4:** Mean ± SD and range of insulin concentrations (mg/ml) in male and female Asian and African elephants.

	Asian			African		
Sex	N	Mean ± SD	Min-Max	N	Mean ± SD	Min-Max
Female	80	1.56 ± 1.20	0.23–6.24	100	0.65 ± 0.52	0.18–3.07
Male	13	0.62 ± 0.50	0.11–1.65	17	0.56 ± 0.49	0.10–1.89
Overall	94	1.42 ± 1.18	0.11–6.24	117	0.64 ± 0.52	0.10–3.07

### Glucose-to-insulin (G:I)

Mean and range of the G:I according to species and sex are presented in Table 5. There was a difference in the G:I between Asian and African elephants overall, with African elephants having nearly double the G:I compared to Asian elephants (P<0.05). There were species differences in the G:I of females; African females had a higher, again nearly double, mean G:I compared to Asian females (P<0.001). There was no difference in mean G:I between African males compared to Asian males (P = 0.172). The lowest G:I value was observed in an Asian female (14), and the highest in an African male elephant (917). Similar to the leptin results, there was a large amount of variation in the G:I data.

**Table 5:** Mean ± SD and range (min-max) of the glucose-to-insulin ratio (G:I) in male and female Asian and African elephants.

	Asian			African		
Sex	N	Mean ± SD	Min-Max	N	Mean ± SD	Min-Max
Female	79	110 ± 86	14–430	100	208 ± 113	30–516
Male	13	253 ± 18	70–603	16	326 ± 23	69–917
Overall	92	130 ± 11	14–603	116	224 ± 140	30–917

### Metabolic hormones associated with health parameters

Relationships between leptin and G:I and health parameters are presented in Tables 6–9. There were significant associations between leptin and cyclicity status. As leptin concentration increased there was an increase in odds of a female being non-cycling. The G:I was associated inversely with body condition, as the G:I increased there was a decreased risk of BCS = 4 or 5 as compared to the ideal, or BCS = 3. Neither leptin nor the G:I were predictive of foot or musculoskeletal health. Demographic variables including Age, Sex, and Species were included in the models as confounders as indicated in the predictive models for each health outcome in Tables 6–9.

**Table 6:** Leptin and the glucose-to-insulin ratio (G:I) in predicting foot health using Poisson regression.

Variable	Beta	Relative Risk	P-Value
Intercept	-0.050	-	0.879
Leptin	-0.016	0.984	0.637
G:I	0.0001	1.000	0.544
Age (confounds G:I and leptin)	0.023	1.022	0.002
Species (confounds G:I)	-0.157	0.890	0.554
Sex (confounds leptin)	0.002	1.002	0.992

**Table 7:** Leptin and the glucose-to-insulin ratio (G:I) in predicting musculoskeletal health using multinomial logistic regression.

Variable	Beta	Odds Ratio	P-Value
Intercept 1	3.364	-	<0.001
Intercept 2	4.480	-	<0.001
Intercept 3	7.482	-	<0.001
Leptin	-0.033	0.973	0.704
G:I	-0.002	0.998	0.444
Age (confounds G:I and leptin)	-0.059	0.942	0.003
Species (confounds G:I)	1.022	2.779	0.198
Sex (confounds G:I and leptin)	-0.054	0.947	0.908

**Table 8:** Leptin and the glucose-to-insulin ratio (G:I) in predicting cyclicity status using repeated measures logistic regression.

Variable	Beta	Odds Ratio	P-Value
Intercept	-4.565	-	0.005
Leptin	0.231	1.259	0.008
G:I	-0.007	0.993	0.754
Age (confounds leptin and G:I)	0.065	1.067	0.055
Species (confounds G:I)	1.800	6.051	0.009

**Table 9:** Leptin and the glucose-to-insulin ratio (G:I) in predicting body condition using multinomial logistic regression.

Variable	Beta	Odds Ratio	P-Value
Intercept 1	-1.509	-	0.009
Intercept 2	0.529	-	0.341
Leptin	0.085	1.089	0.157
G:I	-0.005	0.995	<0.001
Age (confounds leptin)	-0.036	0.964	0.020

### Association of zoo variables with metabolic hormones

A variety of demographic, management, housing, and social zoo variables were investigated as predictors of leptin and G:I levels as shown in Table 10. Significant relationships with leptin were found for: Walk Week (negative), Feed Diversity (positive), Space Experience per Elephant (negative), Animal Contact (negative), and Social Group Contact (negative). For the G:I, significant relationships were found for: Age (negative), Sex (negative), Species (negative), Exercise Week (negative), Walk Week (negative), Feeding Predictability (positive), Feed Diversity (negative), Percent Time Indoor (negative), Space Experience per Elephant (positive), Space Experience (positive), and Relative Positive Reinforcement and Negative Punishment (positive).

**Table 10:** Associations between mean leptin levels and the G:I with zoo variables.

		Leptin			G:I		
Variable	Reference	N	Beta	*P v*alue	N	Beta	*P v*alue
*Demographics*							
* *Age		192	0.020	0.149	208	-2.477	<0.001
Sex	ref = Male	41			29		
	Female	151	-0.201	0.692	179	-128.477	0.0013
Species	ref = African	105			116		
	Asian	87	-0.085	0.828	92	-93.990	<0.001
*Exercise*							
Exercise Week	ref = 1	33			32		
	2	78	0.007	0.989	83	-76.094	0.019
	3	0					
	4	18	1.198	0.315	20	-31.531	0.474
	5	31	-0.669	0.225	35	-117.120	0.001
	6	2	-0.284	0.559	3	-186.780	<0.001
	7	10	0.428	0.531	15	-82.114	0.158
Walk Week	ref = 1	76			81		
	2	6	0.866	0.050*	66	-12.103	0.663
	3	0					
	4	14	-0.036	0.958	15	-11.745	0.824
	5	9	-0.480	0.089*	11	-97.345	<0.001
	6	4	2.097	0.033*	7	37.654	0.608
	7	6	-0.180	0.571	8	-76.720	0.032
*Feeding*							
Feed Day		176	0.043	0.536	194	-4.814	0.180
Feed Night		176	0.110	0.454	194	-4.445	0.485
Feed Total		176	0.040	0.476	194	-3.419	0.173
Feeding Predictability	ref = 1	138			152		
	2	38	-0.137	0.748	42	52.276	0.075
Feed Diversity		176	1.423	0.007*	194	-120.312	0.001
Spread		176	-0.874	0.473	194	94.221	0.263
Alternative Feeding Methods		182	1.373	0.144	194	-27.982	0.556
*Housing*							
Percent Time Indoor		190	-0.003	0.707	206	-0.933	0.071
Percent Time In/Out Choice		190	0.014	0.114	206	0.756	0.289
Space Experience per Elephant		190	-0.009	0.041*	206	1.241	0.046
Space Experience		190	-0.003	0.123	206	0.326	0.056
Time on hard substrate		190	-0.000	0.961	206	-0.208	0.747
Time on soft substrate		190	-0.001	0.907	206	-0.636	0.400
*Social*							
Animal Contact		190	-0.107	0.019*	206	-3.640	0.413
Social Group Contact		190	-0.048	<0.001*	206	-0.514	0.751
Time with Mixed Sex		190	0.001	0.827	206	0.334	0.214
Percent Time Alone		190	0.000	0.940	206	0.562	0.183
*Training and Enrichment*							
Relative Positive Reinforcement and Negative Punishment	Ref = 5	10			12		
	6	26	-0.210	0.828	27	60.888	0.030
	7	66	-0.633	0.485	81	94.049	0.003
	8	64	-0.492	0.595	66	78.439	0.017
	9	2	4.159	0.279	1	-42.333	0.080
Enrichment Diversity		175	-0.174	0.900		-65.203	0.374

### Predictive models

The leptin predictive model indicated that a combination of Walk Week, Feed Diversity, Space Experience per Elephant, and Social Group Contact (P*<*0.05) had the greatest effect on leptin levels (Table 11). The model predicts that leptin will decrease by 0.769 ng/ml if the time spent in staff directed walking is increased from 1 hour/week to 7–10 hours per week; by 0.047 ng/ml with every additional social group; and by 0.0132 ng/ml for every additional 500 ft^2^ of Space Experience per Elephant. Finally, the analysis predicts that elephants with more diverse feeding programs tend to have significantly higher leptin levels. Demographic variables including Age and Sex were included in the model as non-significant confounders. Age confounded Social Group Contact and Space Experience per Elephant, and Sex confounded Space Experience per Elephant in the final predictive model. Descriptive statistics for the significant variables in the leptin multi-model are presented in Table 12.

**Table 11:** Predictive model describing primary variables associated with mean leptin concentration.

Parameter	Estimate	Standard Error	Pr > |Z|	
Intercept	2.280	0.704	0.001	
Age	0.024	0.015	0.117	
Sex: Male	-	-	-	
Sex: Female	-0.531	0.624	0.395	
Social Group Contact	-0.047	0.018	0.007	*
Space Experience Per Elephant (500 ft^2^)	-0.013	0.006	0.034	*
Feed Diversity	1.537	0.471	0.001	*
Walk Week 1: Less than 1 hour per week	-	-	-	
Walk Week 2: Between 1–3 hours per week	0.669	0.418	0.109	
Walk Week 3: Between 4–6 hours per week	-	-	-	
Walk Week 4: Between 5–7 hours per week	-0.477	0.694	0.491	
Walk Week 5: Between 7–10 hours per week	-0.769	0.309	0.013	*
Walk Week 6: Between 10–14 hours per week	1.542	1.075	0.152	
Walk Week 7: 14 or more hours per week	-0.480	0.258	0.037	*

*P < 0.05

**Table 12:** Descriptive statistics for variables in the leptin model.

Variable	N	Mean	SD	Min	Median	Max
Feed Diversity	176	1.37	0.26	0.30	1.40	1.79
Space Experience per Elephant (per 500 ft^2^)	190	23.11	21.79	0.66	16.32	140.09
Social Group Contact	190	4.08	5.58	1	2	30
Walk Week	172	2.15	1.57	1	2	7

The predictive model for the G:I found that a combination of Sex, Species, Percent Time Indoor, Feed Diversity, and Feeding Predictability was significant as shown in Table 13. Females had a mean G:I of 114 units lower than males, and Asian elephants had a G:I of 87 units lower than African elephants. The G:I decreased by 8 units for every 10% increase in the time spent indoors (Percent Time Indoor). Two feeding variables were included in the model, which predicted that elephants with more diverse feeding programs had a significantly lower G:I, and elephants with unpredictable feeding schedules had a G:I of 48 units higher than elephants with a predictable feeding schedule. Analyses did not reveal any confounders in the final model. Descriptive statistics for the significant variables in the G:I multi-model are presented in Table 14.

**Table 13:** Predictive model describing primary variables associated with mean G:I.

Parameter	Estimate	Standard Error	Pr > |Z|	
Intercept	19.242	43.798	0.660	
Species: African	-	-	-	
Species: Asian	-87.387	16.858	<0.001	*
Sex: Male	-	-	-	
Sex: Female	-113.791	42.206	0.007	*
Feed Diversity	-94.871	29.629	0.001	*
Percent Indoor	-0.800	0.419	0.050	*
Feeding Predictability: Predictable	-	-	-	
Feeding Predictability: Unpredictable	47.476	20.358	0.019	*

*P < 0.05

**Table 14:** Descriptive statistics for variables in the G: I model.

Variable	N	Mean	SD	Min	Median	Max
Feed Diversity	194	1.36	0.26	0.304	1.39	1.791
Feeding Predictability: Unpredictable	194	1.22	0.41	1	1	2
Percent Time Indoor	206	27.59	22.58	0	23.97	81.04

## Discussion

Our study is the first large-scale metabolic hormone assessment of zoo Asian and African elephants, and epidemiological analyses of the risk factors associated with metabolic status. Higher leptin levels were associated with ovarian inactivity, and the G:I was predictive of body condition, with a higher BCS being associated with a lower G:I ratio. These results confirm an earlier study that found similar relationships between leptin and G:I with reproductive cyclicity and body condition in a smaller sample of female African elephants [36]. The present study further included an analysis of key factors in the zoo environment that influenced hormone concentrations, and found that walking and feeding practices were most influential in predicting elephant metabolic status. Given well-known adverse consequences of high leptin and low G:I to animal health [17–26], identifying zoo factors that influence metabolic hormone levels and implementing management changes to mitigate negative effects could be crucial to ensuring population sustainability.

The final leptin predictive model indicated that a combination of Walk Week, Feed Diversity, Space Experience per Elephant, and Social Group Contact (P*<*0.05) had the greatest effect on leptin concentrations. More walking was protective against high leptin levels, which parallels findings in numerous other species, where walking has been found to be an effective means of controlling excessive adipose tissue and corresponding leptin secretion [42–44]. For elephants, the benefits of walking were detected if the time spent in staff-directed walking was 7 hours or more per week. The model predicted that leptin would decrease by 0.77 ng/ml if time spent in staff-directed walking increased from 1 hour/week to 7–10 hours per week. Given a mean leptin of 4.12 ng/ml for all study animals, this equates to an approximate 20% decrease in leptin if walking is increased to at least 1 hour per day. Walk Week was also related to BCS with 14 or more hours per week being predictive of a decreased risk of BCS 4 or 5 (overweight elephants) [37]. Thus, as expected, walking in zoo elephants would appear to be good for elephant health, perhaps in part by normalizing serum leptin levels and promoting optimal body condition. By contrast, an increase in Feed Diversity was associated with higher leptin levels, consistent with the negative effects observed between that and both G:I (this study) and BCS [37]. Higher Feed Diversity scores indicate use of a variety of food presentation methods, such as browse, food puzzles, and hanging or hidden food items, in addition to regular trough or floor feeding [41]. Although dietary intake was not monitored, we suspect that more feeding opportunities may be associated with an increase in food presented/consumed and thus calories consumed, which could result in higher adiposity and leptin secretion. This emphasizes the importance of factoring in food enrichment in the overall dietary plan of zoo elephants to properly control caloric intake. Investigations designed to test this relationship are warranted, especially given the important role and prevalence of enrichment in elephant care practices.

The variable Space Experience per Elephant can be used to reflect elephant density [39], and results suggest a lower density was associated with lower leptin levels. This variable considered the total space experience, combining both indoor and outdoor environments, divided by the total number of elephants using that space. In a study of mice using social crowding as a chronic stress model, serum leptin was elevated in association with increased adiposity, demonstrating stress-related metabolic dysfunction [45]. Stress-induced glucocorticoids can stimulate leptin synthesis and secretion [46–48]. In our study, the magnitude of effect was small, such that Space Experience per Elephant increases of 5,000 ft^2^ were predicted to be associated with only a 0.13 ng/ml decrease in leptin. Thus, using the population mean Space Experience per Elephant of 11,555 ft^2^, doubling this space would only decrease leptin by 0.31 ng/ml, a reduction of approximately 13% from the population mean. Thus, implementing ways to increase Space Experience per Elephant might be beneficial to metabolic health, but only if the increase in space was substantial. Otherwise, focusing on walking and feeding factors may be more practical to elicit changes in leptin concentrations if needed.

A social variable, Social Group Contact, was significant in the multi-variable leptin model. This variable quantifies the common management practice of dividing elephants into sub-groups with individual elephants spending time in one or more of these social configurations [39]. As Social Group Contact increased, leptin concentrations decreased. Increased social withdrawal and depressive symptoms are correlated with increased leptin levels in humans [49, 50], suggesting being exposed to more social groups may have a positive effect on leptin. By contrast, this variable was positively associated with hyperprolactinemia in female African elephants, a suspected cause of infertility in that species as it is in other species, and a physiological response to social stress [32]. Thus, we cannot rule out that lower leptin levels in association with increased Social Group Contact may be associated with depressive disorders or anxiety, similar to that described in response to social stress in humans and animal models [45, 51]. Leptin signaling is involved in the pathophysiology of major depressive disorders, exerting its effects by activation the leptin receptor, which is distributed in various brain regions such as the prefrontal cortex and hippocampus, two limbic brain areas implicated in depression [49]. Because we found increased leptin was associated with acyclicity in this and another study [36], and high body condition was also related to increased leptin [36], it is suspected that strategies aimed at decreasing leptin, such as promoting compatible social groupings, would promote healthier elephants. However, again, this effect was small. The model predicts that leptin will decrease 0.047 ng/ml for each additional social group, so it would require an additional 10 groups to decrease leptin by 0.47 ng/ml. Using data from Morfeld and Brown [36], comparing leptin concentrations in non-cycling and cycling African females, it would take an additional 25 social group contacts to reduce leptin in non-cycling elephants to that of the cycling group; i.e., reduce concentrations from 4.75 to 3.58 ng/ml, which would be very difficult to implement. The contrasting results in regard to social groups on hyperprolactinemia and leptin highlight the complexity of social variables on hormone levels, and certainly warrant further investigation.

Several factors were significant predictors of G:I and could be targeted to address problems associated with excessive body condition and reproductive acyclicity. Two feeding variables were significantly related to G:I: Feeding Predictability and Feed Diversity. The schedule of feedings (Feeding Predictability) impacted G:I status, with implementation of a random rather than predictable feeding schedule being associated with increased, or healthier, G:I. Elephants were predicted to have 47 units higher G:I if feeding was random, which is a substantial effect given the overall mean G:I for all study animals was 182. In humans, it is now recognized that varying the *timing* of food intake can be a simple method for preventing metabolic dysfunction and obesity [52]; our results suggest the same may be true for elephants. Higher insulin levels were observed in humans when food was rationed into a small number of predictable feedings, compared to higher frequency, random feedings [53, 54]. High insulin concentrations inhibit lipase enzyme activity and increase fat deposition, thus resulting in excess adipose tissue and leptin levels [55]. In elephants, low G:I (this study) and elevated serum insulin concentrations [36] are characteristic of elephants with high BCSs, so a similar mechanism involving insulin regulation may contribute to excessive fat, and higher leptin levels, in zoo elephants. Offering food more randomly could have an effect on normalizing serum metabolic hormones, and hence promote healthier elephants.

In addition to being associated with higher leptin, we found that higher Feed Diversity was associated with a lower, or unhealthy G:I. It is possible that more diverse feeding methods also equated to additional food items being consumed, including those with a high glycemic index, and thus resulted in a lower G:I. The glycemic index refers to a measure of a food’s carbohydrate impact during digestion and its conversion into glucose. Foods with a higher score have the most impact on acute and chronic blood glucose levels, resulting in metabolic hormone dysregulation and weight gain [56, 57]. This is supported by higher BCSs indicative of overweight and obesity in elephants with higher Feed Diversity scores [37]. Thus, choosing foods with a lower glycemic index may promote optimal glucose and insulin regulation and body fat. Feed Diversity was also found to be related to mean daily walking distances [58]; greater mean daily walking distances with higher Feed Diversity, with authors proposing that elephants who experience more feeding methods are more likely to explore their environments in search of them, and as such walk greater distances. However, there was not a relationship between mean daily walking distance and BCS [58]. It is possible that the calories required to access the additional feeding methods was not sufficient to reduce their effect on body condition [37]. It is important to note that data was not collected on the *types or quantity* of foodstuffs presented or consumed, but rather focused on the non-nutritional aspects of elephant feeding programs to determine the patterns of food provision utilized across zoos and the degree of diversity in the food delivery methods employed [41]. A logical next step would be to investigate both the nutritional and non-nutritional components of feeding programs and how they affect metabolic status and related conditions.

A housing variable, Percent Indoor, was related to G:I, with more time spent indoors being predictive of a lower G:I. With each 10% increase in time spent indoors, elephants had an approximately 8 unit decrease in G:I. This effect is likely due to the generally smaller size of indoor compared to outdoors spaces [39], which may limit locomotion and its protective effects on insulin and glucose regulation [59, 60]. Holdgate et al. [58] assessed voluntary walking distance in relationship to BSC and found no relationship; however, the study only assessed outdoor walking distance. There also could be negative effects on metabolic regulation in situations of overcrowding, like that described for mice [45], which is more likely to occur in indoor space.

Overall, we found that metabolic hormones are influenced most significantly by walking and feeding practices, and are related to fitness parameters including reproductive cyclicity and body condition. It is important to continue exploring the relationship between metabolic hormones, health issues, zoo factors and their impact on elephant welfare. The methods we use to monitor leptin, glucose, and insulin are now also available for comparing levels of zoo elephants to those of free-ranging elephants. To obtain comparative data from free-ranging elephants, Morfeld (KM) has recently established a field program and laboratory in South Africa. Morfeld also completed a research project in 2016 assessing insulin, glucose, and cortisol concentrations in 180 elephant serum samples (male and female, variety of age classes) with South Africa’s Kruger National Park, Veterinary Wildlife Services. Morfeld’s research programs in South Africa aim to obtain comparative data from semi- and free-ranging elephants for comparison to zoo-managed elephants. This data will be used to inform management practices in both captive and free-ranging elephants to promote global population sustainability.

We suggest continual longitudinal monitoring of metabolic hormones to provide insight into metabolic health of individual elephants. For example, elephants at risk for ovarian acyclicity or high body condition could potentially be identified in time for preventative measures to be implemented. Furthermore, hormone monitoring could be extremely useful in determining if a change in management aimed at addressing a health issue is in fact effective, or if modifications should be made. To best utilize the findings of this study, zoos should assess their elephant program to determine how they can promote random feedings, implement walking a minimum of 7 hours per week (or 1 hour per day), limit the diversity of food presentation methods (or at least using lower calorie or low glycemic foodstuffs in the food presentation methods), limit time spent indoors, and promote utilization of all available space. Given the uniqueness and complexity of individual zoo programs, zoos must be creative in how they implement these recommendations so that management strategies promote good metabolic health and thus welfare of elephants in their care.
